# Identifying Non-Linear Association Between Maternal Free Thyroxine and Risk of Preterm Delivery by a Machine Learning Model

**DOI:** 10.3389/fendo.2022.817595

**Published:** 2022-02-24

**Authors:** Yulai Zhou, Yindi Liu, Yuan Zhang, Yong Zhang, Weibin Wu, Jianxia Fan

**Affiliations:** ^1^ The International Peace Maternity and Child Health Hospital, Shanghai Jiao Tong University School of Medicine, Shanghai, China; ^2^ Shanghai Key Laboratory of Embryo Original Diseases, Shanghai, China; ^3^ Shanghai Municipal Key Clinical Specialty, Shanghai, China

**Keywords:** free thyroxine, spontaneous preterm delivery, iatrogenic preterm delivery, Isolated hypothyroxinemia, overt hyperthyroidism, generalized additive model

## Abstract

**Objective:**

Preterm delivery (PTD) is the primary cause of mortality in infants. Mounting evidence indicates that thyroid dysfunction might be associated with an increased risk of PTD, but the dose-dependent association between the continuous spectrum maternal free thyroxine (FT4) and PTD is still not well-defined. This study aimed to further investigate this relationship using a machine learning-based model.

**Methods:**

A hospital-based cohort study was conducted from January 2014 to December 2018 in Shanghai, China. Pregnant women who delivered singleton live births and had first-trimester thyroid function data available were included. The generalized additive models with penalized cubic regression spline were applied to explore the non-linear association between maternal FT4 and risk of PTD and also subtypes of PTD. The time-to-event method and multivariable Cox proportional hazard model were further applied to analyze the association of abnormally high and low maternal FT4 concentrations with the timing of PTD.

**Results:**

A total of 65,565 singleton pregnancies with completed medical records and no known thyroid disease before pregnancy were included for final analyses. There was a U-shaped dose-dependent relationship between maternal FT4 in the first trimester and PTD (*p <*0.001). Compared with the normal range of maternal FT4, increased risk of PTD was identified in both low maternal FT4 (<11.7 pmol/L; adjusted hazard ratio [HR] 1.34, 95% CI [1.13–1.59]) and high maternal FT4 (>19.7 pmol/L; HR 1.41, 95% CI [1.13–1.76]). The association between isolated hypothyroxinemia and PTD was mainly associated with spontaneous PTD (HR 1.33, 95% CI [1.11–1.59]) while overt hyperthyroidism may be attributable to iatrogenic PTD (HR 1.51, 95% CI [1.18–1.92]) when compared with euthyroid women. Additionally, mediation analysis identified that an estimated 11.80% of the association between overt hyperthyroidism and iatrogenic PTD risk was mediated *via* the occurrence of hypertensive disorders in pregnancy (*p <*0.001).

**Conclusions:**

We revealed a U-shaped association between maternal FT4 and PTD for the first time, exceeding the clinical definition of maternal thyroid function test abnormalities. Our findings provide insights towards the need to establish optimal range of maternal FT4 concentrations for preventing adverse outcomes in pregnancy.

## Introduction

Preterm delivery (PTD, also acknowledged as preterm birth) is the primary cause of mortality in neonates, infants, and also younger children, and is defined as any live birth before 37 completed weeks of pregnancy (1–3). PTD annually affects ~15 million newborns globally with 1.2 million (7.8%) in China (4). Furthermore, preterm birth children are at increased risk of serious illness (e.g., lung immaturity, infection) and are susceptive to neurodevelopmental, cognitive, cardiovascular, and metabolic disorders in adulthood (5–9). Although several risk factors for PTD have been reported (e.g., history of PTD, advanced maternal age, low socioeconomic status, exposure to smoking or narcotics) (1, 10), the mechanisms that lead to PTD is still not understood. Moreover, the relationship between maternal thyroid function and PTD has not been fully elucidated.

Maternal thyroid hormones (namely, thyroxine or tetraiodothyronine [T4] and triiodothyronine [T3]) in early pregnancy are associated with intra-uterine inflammation, placentation functions, and adverse pregnancy complications (e.g., intrauterine growth retardation and pre-eclampsia) (11, 12). There is mounting evidence indicating that an increased risk of PTD might be related to both maternal hyper- and hypothyroidism (13, 14). A study in 2015 demonstrated a statistically significant incremental risk of PTD among pregnant women with overt hyper- and hypothyroidism while not in women with mild thyroid dysfunction (e.g., subclinical hypothyroidism or isolated hypothyroxinemia) (15). Moreover, it was revealed that women with isolated hypothyroxinemia in early pregnancy had a higher risk of spontaneous PTD (16). In contrast, recent research illustrated that higher maternal free thyroxine (FT4) concentration was associated with a reduced risk of PTD under a linear regression model (17). In this study, we proposed that there might be a non-linear shape of the dose–response relationship between FT4 and the risk of PTD, which have not been established until recently.

Therefore, the primary goal of this study was to evaluate the non-linear association between the continuous spectrum of maternal FT4 concentrations with the risk of PTD and its subtypes by a machine learning-based model.

## Materials and Methods

### Study Population

Pregnant women who delivered between January 2014 and December 2018, with records of first-trimester antenatal screening and regular antenatal visits at the International Peace Maternity and Child Health Hospital (IPMCH), a tertiary university-attached maternity center in Shanghai, China, were included. Written informed consent was obtained from all participants when they registered in the hospital. Exclusion criteria were: fetal chromosome abnormality, multiple pregnancies, *in vitro* fertilization, miscarriage, fetal death, diabetes or hypertension before pregnancy, or a history of either thyroid disease or thyroid treatment. Women without available records of thyroid function measurements in the first trimester were also excluded. The study protocol was approved by the Institutional Medical Ethics Committee of IPMCH (GKLW2019-43) and registered at the Chinese Clinical Trial Registry (ChiCTR2000034742).

### Data Collection and Measurement

Data were collected by nurses and gynecologists during routine prenatal pregnancy examinations. This included maternal age, education level, last menstrual period (LMP), parity, and medical history routinely gathered *via* face-to-face interviews during the first antenatal visit. The calculation of pre-pregnant body mass index (BMI) was obtained by dividing the self-reported weight of the patient before pregnancy (in kg) by the square of their height measured by the nurses (in m). Gestational age was estimated by LMP and later adjusted in accordance with ultrasonography results in early pregnancy. Alcohol consumption and smoking status were not included in the analysis as their use were rare (<1%) among pregnant women in our study population.

Quantitative analyses of FT4, thyrotropin (also known as thyroid-stimulating hormone, TSH), and thyroid peroxidase antibody (TPO-Ab) concentrations in fasting blood samples were determined with kits (ARCHITECT i2000; Abbott, Chicago, IL, USA) in accordance with the manufacturer’s protocol in the standardized clinical laboratory of the hospital. The intra- and inter-assay coefficients of variation were, respectively, 1.6 and 3.59% for TSH; 1.9 and 4.01% for FT4; and both 10% for TPO-Ab. TPO-Ab concentrations exceeding 5.6 IU/ml was considered positive per the cut-off value defined by the manufacturer. Data were extracted from the medical record system of the hospital by experienced information engineers.

### Diagnostic Criteria and Outcomes

The local population-based reference range (P2.5–P97.5) of FT4 and TSH in early pregnancy is 11.7–19.7 pmol/L and 0.03–3.64 mIU/L, respectively. According to the reference ranges, we defined overt hypothyroidism as FT4 <P2.5 with TSH >P97.5; isolated hypothyroxinemia as FT4 <P2.5 with TSH within normal range; overt hyperthyroidism as FT4 >P97.5 with TSH <P2.5; subclinical hyperthyroidism as TSH <P2.5 with FT4 within the normal range; subclinical hypothyroidism as TSH >P97.5 with FT4 within the normal range; and isolated hyperthyroxinemia as FT4 >P97.5 with TSH within the normal range.

Gestational diabetes mellitus (GDM) was diagnosed in a 2-h 75 g oral glucose tolerance test at 24–28 weeks of pregnancy according to the criteria of the American Diabetes Association (18).

Hypertensive disorders in pregnancy (HDP) included gestational hypertension and pre-eclampsia, diagnosed by blood pressure measurements ≥140 mmHg systolic or 90 mmHg diastolic at least twice within 4–6 h, with or without proteinuria. The proteinuria was defined as ≥300 mg protein in a 24-h urine sample or a urine dipstick positive test (19).

The primary outcome was PTD, defined as birth before 37 weeks of pregnancy. The secondary outcomes were the PTD subtypes, namely, spontaneous PTD (defined as the spontaneous onset of labor with intact membranes or after preterm premature rupture of the membranes) and iatrogenic PTD (defined as labor induction with intact membranes or by C-section delivery without labor due to maternal or fetal indications) (1).

### Statistical Analyses

Continuous variables with normal distribution were presented as mean ± standard deviation (SD), and non-normally distributed variables were shown as medians with interquartile range (IQR). Categorical variables were demonstrated as numbers (percentages) for baseline characteristics.

The generalized additive model (GAM) with penalized cubic regression spline (*k* = 5) were applied to explore a smooth, potential non-linear association between maternal FT4 and risk of PTD and the duration of gestation, allowing a better fit than models assuming a strict linear association (20, 21). For GAM analyses, continuous TSH and FT4 concentrations were analyzed after the removal of outliners (0.5%).

For time-to-event analyses, time was determined as the gestational weeks at delivery, PTD as an event, and a term or post-term delivery was censored at delivery. To estimate the adjusted accumulative incidence of PTD, a three-category maternal FT4-categorized Kaplan–Meier estimate of the probability of PTD was appraised with variances reported from log-rank tests overall and pairwise between subgroups, after adjustment for multiple comparisons *via* the Benjamini & Hochberg method.

To further compare the risk of PTD across different types of thyroid test abnormalities, a multivariable Cox proportional hazards regression model was conducted after adjusting for maternal age, education level, medical insurance status, parity, fetal sex, pre-pregnant BMI, and TPO-Ab. The potential confounders were chosen based on the biological plausibility, the selection of confounders in the previous studies, and changes of the effect estimate of interest. Mediation analysis was employed to determine potential mediation effects of HDP on the association of FT4 with iatrogenic PTD. The total effect of FT4 on iatrogenic PTD was divided into average direct effects (ADEs) and the average causal mediation effects (ACMEs)—the effect mediated *via* the development of HDP (22). The mediation proportion was estimated as the ACMEs divided by the total effect.

Based on previous studies concerning the risk factors for PTD (2, 10, 17), their association with FT4 (23–25), and also their clinical relevance, we further stratified pregnant women into the following subgroups: (1) age <35- or ≥35-year-old groups; (2) pre-pregnant BMI <18.5, 18.5–23.9, or ≥24 kg/m^2^ groups in accordance with the Chinses Working Group on Obesity (26); (3) TPO-Ab positive or -negative groups; and (4) nulliparity or multiparity groups—to assess whether the findings were affected by advanced age, abnormal BMI, thyroid autoimmunity, or multiparity. Sensitivity analyses were conducted to assess the robustness of results excluding women with either a history of PTD, thyroid medication use during pregnancy, GDM, or HDP.

All analyses were conducted with R Software v3.6.3 (R Project for Statistical Computing; with packages *mgcv*, *ggplot2*, *forestplot*, *survminer*, *survival*, and *mediation*). Statistical significance was set at *p <*0.05 (all tests were 2-sided).

## Results

### Population Characteristics

**Figure 1:**
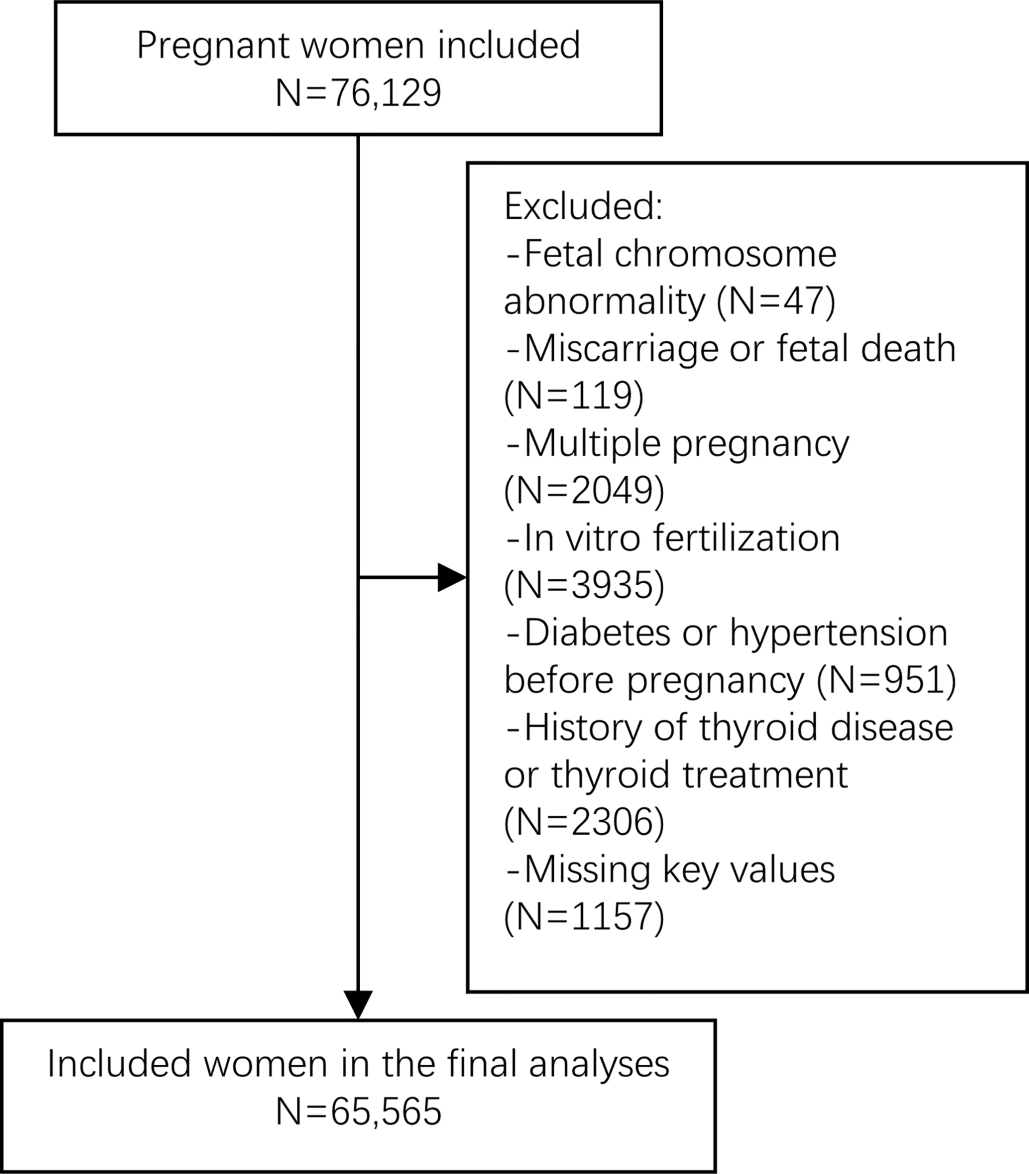
Flow chart of the study population.

The final study population comprised 65,565 pregnant women (**Figure 1**). The baseline population characteristics are presented in **Table 1**. The mean maternal age was 30.5 ± 3.77 years. The median of gestational weeks at delivery was 39.1 (38.4–40.0) weeks. The study population was mainly primiparous 48,383 (73.8%), mostly normal weight 47,833 (73.0%) with a pre-pregnant BMI of 18.5–23.9 kg/m^2^, and a low ratio of PTD history 771 (1.18%). The median weeks of maternal thyroid function examined was 12.1 (11.7–12.6). The median of TSH and FT4 in the first trimester were 1.18 (0.65–1.84) mIU/L and 14.3 (13.3–15.5) pmol/L, respectively. The percentage of TPO-Ab positivity was 7,097 (10.8%). The missing variables included 528 (0.8%) entries of education level, which was missing at random.

**Table 1:** Demographic data of the study population (N = 65,565).

Characteristics	Participants, No. (%)
Maternal characteristics	
Age, mean (SD), y	30.5 (3.77)
Prepregnant BMI^a^	
<18.5	9,794 (14.9%)
18.5–23.9	47,833 (73.0%)
≥24	7,938 (12.1%)
Primiparous	48,383 (73.8%)
Education Level	
High school and below	4,445 (6.8%)
College	47,882 (73.0%)
Postgraduate	12,710 (19.4%)
Missing	528 (0.8%)
Insurance	51,202 (78.1%)
History of preterm delivery	771 (1.18%)
Hypertensive disorders in pregnancy	2,961 (4.5%)
Gestational diabetes	8,004 (12.2%)
FT4, median (IQR), pmol/Lb	14.3 (13.3, 15.5)
TSH, median (IQR), mIU/L	1.18 (0.65, 1.84)
TPOAb positive	7,097 (10.8%)
Gestational age for thyroid function test, median (IQR), wk	12.1 (11.7, 12.6)
Fetal characteristics	
Gestational age at birth, median (IQR), wk	39.1 (38.4, 40.0)
Preterm birth (gestational age <37 wk)	3176 (4.8%)
Spontaneous preterm birth	2,127 (3.2%)
Iatrogenic preterm birth	1,049 (1.6%)
Birth weight, mean (SD), g	3,340 (431)
Male sex	33,826 (51.6%)
Female sex	31,739 (48.4%)

Gestational weeks for TBA/thyroid function screening were 9–13 weeks in early pregnancy. BMI, body mass index; IQR, interquartile range; TSH, thyroid-stimulating hormone or thyrotropin; FT4, free thyroxine; TPO-Ab, thyroid peroxidase antibody; SD, Standard deviation.

a Calculated as weight in kilograms divided by height in meters squared.

### Non-Linear Association of Maternal FT4 With Risk of PTD

**Figure 2:**
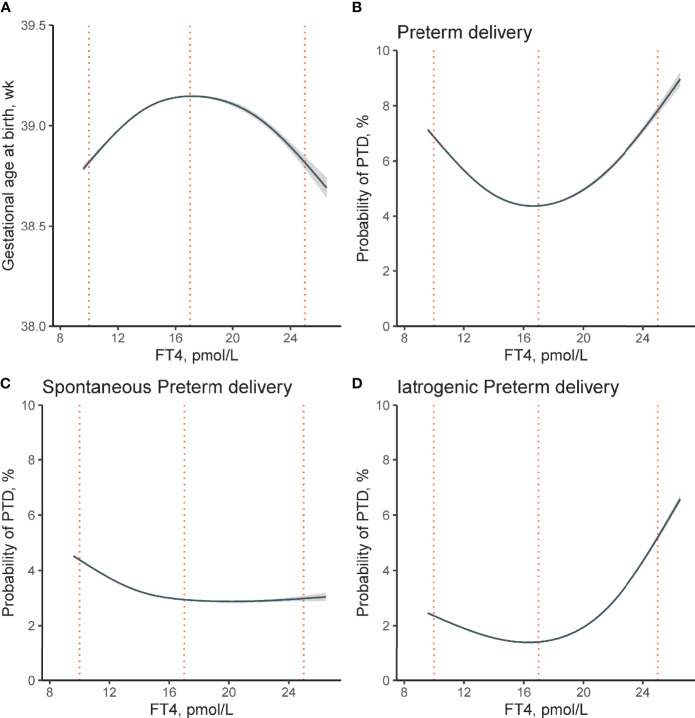
Non-linear association between maternal FT4 concentration in early pregnancy and risk of PTD. Non-linear association between maternal FT4 concentrations and **(A)** gestational age at birth (*χ* ^2^ = 5.18, *p <*0.001), **(B)** the risk of overall PTD (*χ^2^* = 16.36, *p <*0.001), **(C)** spontaneous PTD (*χ^2^* = 6.32, *p* = 0.06), and **(D)** iatrogenic PTD (*χ^2^* = 24.9, *p <*0.001) were analyzed, respectively. The generalized additive models were conducted by adjusting for maternal age, pre-pregnant body mass index, parity, education levels, insurance, TPO-Ab status, and fetal sex. The solid lines and shaded areas represent the estimated mean risk and 95% confidence intervals; dashed vertical lines indicate FT4 concentrations at 10, 17, and 25 pmol/L respectively. FT4, free thyroxine; PTD, preterm delivery; TPO-Ab, thyroid peroxidase antibody.

**Figure 3:**
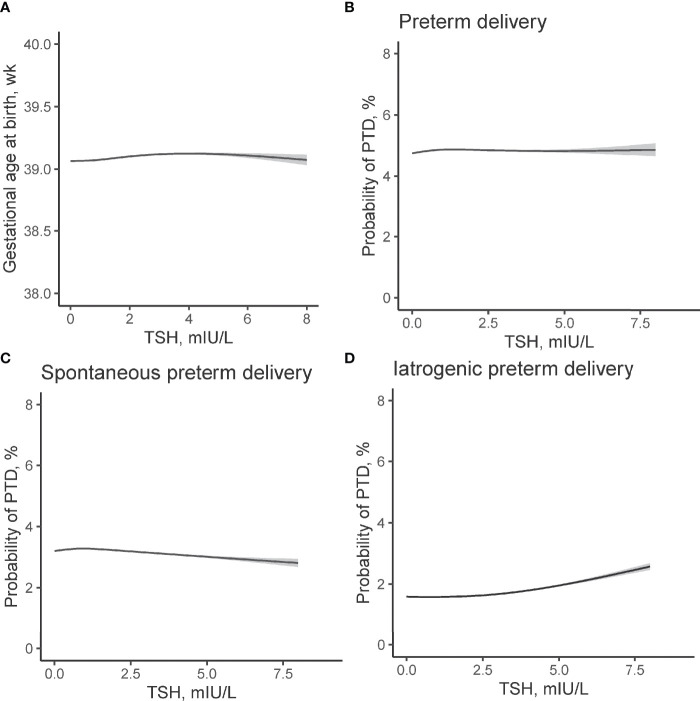
Non-linear association between TSH concentrations in early pregnancy and risk of PTD. Association between maternal TSH concentrations and **(A)** gestational age at birth (χ^2^ = 3.17, *p* = 0.044), **(B)** overall PTD (*χ^2^* = 0.001, *p* = 0.996), **(C)** spontaneous PTD (*χ^2^* = 0.57, *p* = 0.46), and **(D)** iatrogenic PTD (*χ^2^* = 1.67, *p* = 0.34) were evaluated by generalized additive models. The models have 4 *df* and were adjusted for maternal age, fetal sex, pre-pregnant body mass index, parity, education levels, TPO-Ab status, and insurance. The solid lines and shaded areas represent the estimated values and their corresponding 95% confidence intervals. TSH, thyroid-stimulating hormone; PTD, preterm delivery; TPO-Ab, thyroid peroxidase antibody.

Using GAM models, we identified an inverted U-shaped association between maternal FT4 and gestational age at delivery (**Figure 2A**) while no such association was identified between maternal TSH and gestational weeks at birth (**Figure 3A** **)**. The estimated smooth effect curves demonstrating the associations between maternal FT4 and PTD (*p <*0.001), spontaneous PTD (*p* = 0.06), and iatrogenic PTD (*p <*0.001) are shown respectively in **Figures 2B–C**. The fully adjusted smooth curve fitting demonstrated a non-linear U-shaped association between FT4 and PTD (**Figure 2B**), while no such association was observed between maternal TSH and PTD (**Figures 3B–D**).

A significantly increased probability of overall PTD (**Figure 2B**) was observed at both abnormally high and low FT4 concentrations (estimated prevalence of PTD for participants with low FT4 of 10 pmol/L: 6.88%; 95% confidence interval (CI) [6.82–6.94%]; and high FT4 levels of 25 pmol/L: 7.82%; 95% CI [7.67–7.98%]; while the estimated probability of PTD reaches its lowest point at FT4 concentration of 17 pmol/L: 4.38%; 95% CI [4.36–40%]). **Figures 2C, D** further demonstrated the estimated smooth effect curves of the estimated risk of spontaneous and iatrogenic PTD, respectively. In **Figure 2C**, participants with low FT4 of 10 pmol/L tend to have higher estimated risk of spontaneous PTD (4.38%; 95% CI [4.34–4.42%]) when compared with participants with higher FT4. **Figure 2D** showed a steep increase in the estimated risk of iatrogenic PTD among participants of high FT4 of 25 pmol/L (5.21%; 95% CI [5.12–5.29%]) when compared with participants with low FT4 of 10 pmol/L (2.36%; 95% CI [2.33–2.39%]).

### Both High and Low Levels of Maternal FT4 Were Associated With Increased Risk of PTD

**Figure 4:**
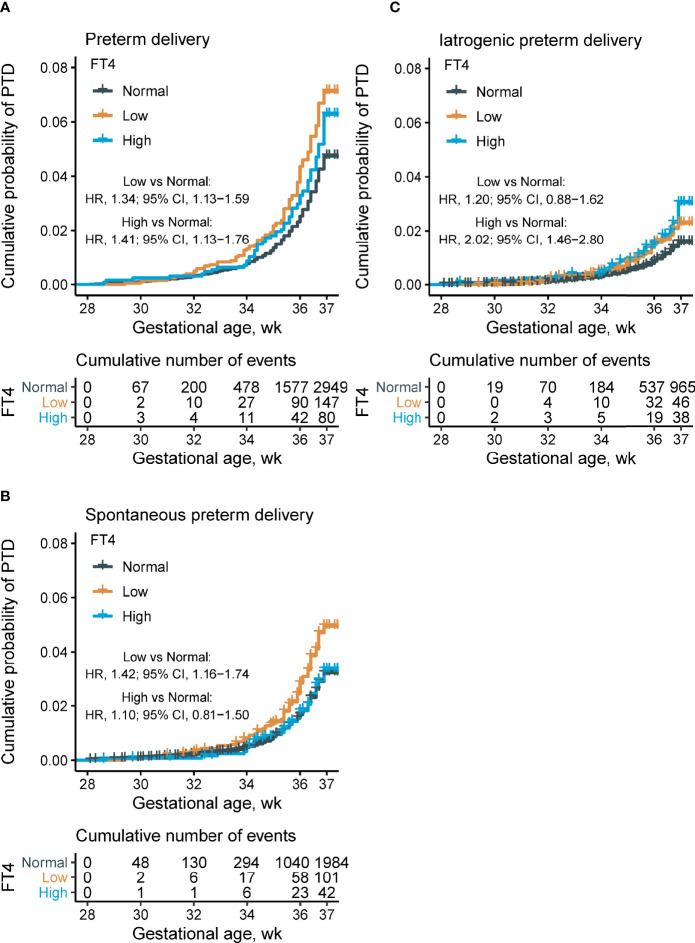
Proportions of overall, spontaneous, and iatrogenic PTD, and time-to-event analysis, by maternal FT4 concentrations. Kaplan–Meier plots showing the proportion of overall PTD **(A)**, spontaneous PTD **(B)**, and iatrogenic PTD **(C)** by different maternal FT4 concentrations categories (low maternal FT4 (<11.7 pmol/L), *n* = 62,227; normal maternal FT4 (11.7–19.7 pmol/L), *n* = 2,064; and high maternal FT4 (>19.7 pmol/L), *n* = 1,274). Cox multivariant analysis was conducted to calculate the hazard ratio by adjusting for maternal age, pre-pregnant body mass index, parity, education levels, insurance, TPO-Ab status, and fetal sex. FT4, free thyroxine; PTD, preterm delivery; TPO-Ab, thyroid peroxidase antibody.

**Figure 5:**
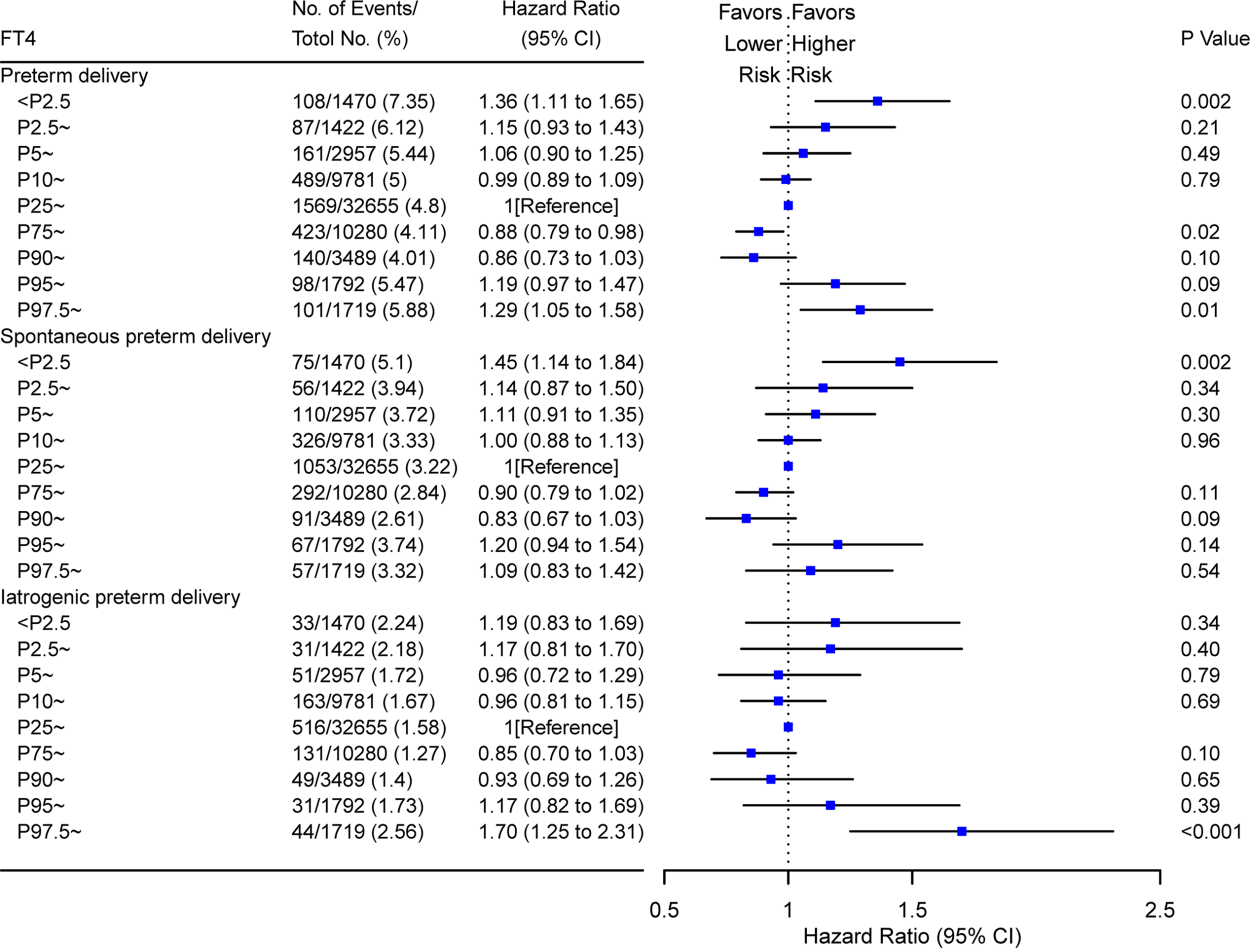
Forest plots for risk of PTD in women with different FT4 percentile at early pregnancy. Hazard ratios of overall PTD, spontaneous PTD, and iatrogenic PTD were shown for FT4 at different percentiles (from low to high). Women with P25–P75 of FT4 were used as the reference control. Model adjusted for maternal age, fetal sex, prepregnant BMI, parity, education levels, TPO-Ab status and insurance; PTD, preterm delivery. FT4, free thyroxine; PTD, preterm delivery; TPO-Ab, thyroid peroxidase antibody.

A time-to-event analysis by gestational week for different FT4 categories showed that the hazard ratio (HR) for PTD in women with FT4 <11.7 and >19.7 pmol/L were significant when compared to women with normal range FT4 concentrations in **Figure 4**. Compared with the normal range of maternal FT4, both low FT4 (<11.7 pmol/L) and high maternal FT4 (>19.7 pmol/L) were significantly associated with 34 and 41% increased risk of PTD, respectively (for low FT4: HR 1.34, 95% CI [1.13–1.59]; for high FT4: HR 1.41, 95% CI [1.13–1.76]). Increasing HRs for spontaneous PTD by gestational week were seen in women with low FT4 (HR 1.42, 95% CI [1.16–1.74]) while the prevalence of iatrogenic PTD was high among women with high FT4 (HR 2.02, 95% CI [1.46–2.80]) after adjusting for confounders. These results were further verified in the stratified analyses, showing 36% (HR 1.36; 95% CI [1.11–1.65]) and 29% (HR 1.29; 95% CI [1.05–1.58]) reduction of PTD risk in women with FT4 <P2.5 and FT4 >P97.5, respectively (**Figure 5**).

### The Association Between Maternal Thyroid Test Abnormalities and Risk of PTD

**Figure 6:**
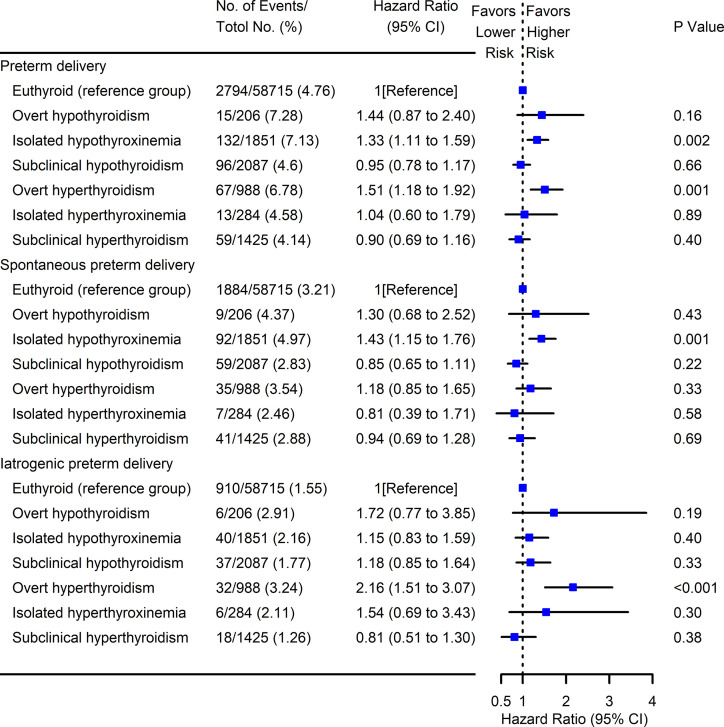
Forest plots for risk of PTD with maternal thyroid test abnormalities. Cox multivariant analysis was conducted for the risk of PTD subtypes (overall, spontaneous, and iatrogenic) with different maternal thyroid test abnormalities by adjusting for maternal age, pre-pregnant body mass index, parity, education levels, insurance, TPO-Ab status, and fetal sex. Pregnancies with euthyroid were used as the reference group. PTD, preterm delivery; TPO-Ab, thyroid peroxidase antibody.

We further explored the association between maternal thyroid test abnormalities and PTD (**Figure 6** **)**. Compared with euthyroid women, an incremental risk of overall PTD was observed among pregnant women with isolated hypothyroxinemia (HR 1.33, 95% CI [1.11–1.59]) and overt hyperthyroidism (HR 1.51, 95% CI [1.18–1.92]) in early pregnancy. Consistent with the results of three groups categorized by maternal FT4, we also found there was a significant association between isolated hypothyroxinemia and spontaneous PTD (HR 1.43, 95% CI [1.15–1.76]), while overt hyperthyroidism and iatrogenic PTD (HR 2.16, 95% CI [1.51–3.07]).

### The Mediating Effect of HDP on the Association Between High FT4 or Overt Hyperthyroidism

**Figure 7:**
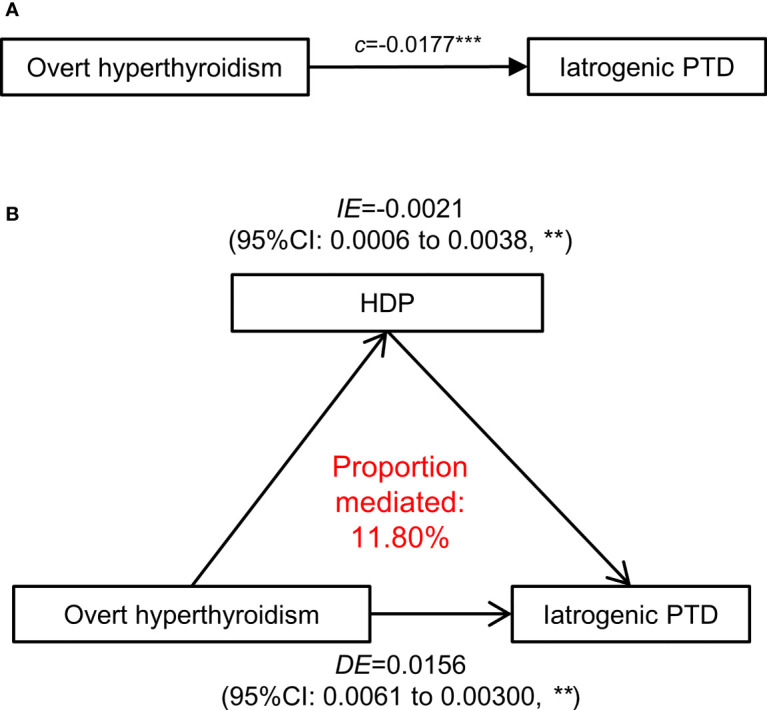
Mediation effect of hypertensive disorders in pregnancy on the association of overt hyperthyroidism with iatrogenic PTD. **(A)** The total effect of maternal overt hyperthyroidism (defined as FT4 >19.7 pmol/L)-iatrogenic PTD relationship was presented as path (c). **(B)** The direct effect in a mediation model is presented and measures the effect of overt hyperthyroidism on the iatrogenic PTD through independent of HDP (the mediator). The difference between Indirect effect and direct effect indicates the effect of maternal overt hyperthyroidism on iatrogenic PTD that operates through development of HDP. HDP, Hypertensive disorders in pregnancy; PTD, preterm delivery; ACME, average causal mediation effects; ADE, average direct effects. All the association adjusted for maternal age, fetal sex, pre-pregnancy BMI, parity, education levels, TPO-Ab status and insurance. The p-values were adjusted for multiple comparisons using the Benjamini & Hochberg method. **p-value < 0.01, ***p-value <0.001.

The mediation analysis unraveled potential mediating effects of HDP on the association between either high FT4 or overt hyperthyroidism and iatrogenic PTD (**Table 2** and **Figure 7**).

**Table 2:** Mediation effect of hypertensive disorders in pregnancy on the association of high-low FT4 concentrations/thyroid dysfunction with iatrogenic/spontaneous PTD.

Total effect (95% CI)	ADE (95% CI)	ACME (95% CI)	Proportion of Mediation (%)
High FT4, HDP and iatrogenic PTD			
0.0169 (0.0063, 0.0300)***	0.0152 (0.0048, 0.0275)***	0.0017 (0.0005, 0.0031)***	10.71 (3.72,19.53)***
Overt hyperthyroidism, HDP and iatrogenic PTD			
0.0177 (0.0077, 0.0325)***	0.0156 (0.0061, 0.0300)***	0.0021 (0.0006, 0.0038)***	11.80 (3.98,25.89)***
Low FT4, HDP, and spontaneous PTD			
0.0127 (0.0049, 0.0200)***	0.0128 (0.005 0.0200)***	−0.0001 (−0.0003, 0.00)	−0.74 (−2.35,0.00)
Isolated hypothyroxinemia, HDP and spontaneous PTD			
0.0137 (0.0051, 0.0200)***	0.0138 (0.0053, 0.0200)***	0.0001 (0.0003, 0.00)	−0.90 (−3.44,0.00)

FT4, free thyroxine; PTD, preterm delivery; ACME, average causal mediation effects; ADE, average direct effects.

FT4 >19.7 pmol/L was categorized as high FT4 and low FT4 was diagnosed with <11.7 pmol/L. Multivariable logistic models were adjusted for maternal age, fetal sex, pre-pregnancy BMI, parity, education levels, TPO-Ab status and insurance.

***p-value <0.001.

The total effect of overt hyperthyroidism on iatrogenic PTD was 0.0177 (95% CI [0.0077–0.0325], *p <*0.001), including a direct mean effect of 0.0156 (95% CI [0.0061–0.0300] *p <*0.001). A mediation effect of overt hyperthyroidism associated with iatrogenic PTD (mean causal mediation effect, 0.0021; 95% CI [0.0006–0.0038], *p <*0.001) through HDP was found, and the estimated proportion of mediation effect was 11.80% (95% CI [3.98–25.89%], *p <*0.001). No mediation effect of HDP was identified in the association between low FT4 or isolated hypothyroxinemia and spontaneous PTD.

We also explored the mediating effect of ICP and placental abruption on the association between maternal FT4 and PTD, and found no such effect (p >0.05, data not shown).

### Subgroup and Sensitivity Analyses

**Figure 8:**
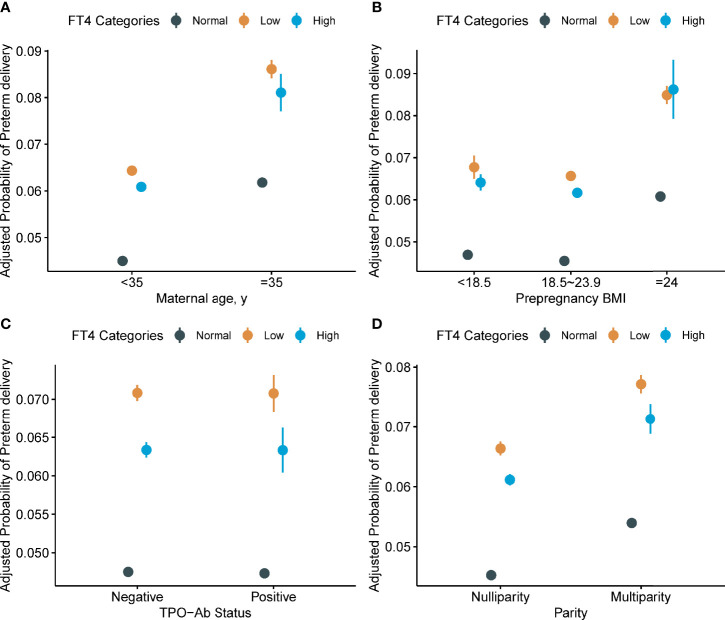
Adjusted probability of preterm delivery (PTD) in different free thyroxine (FT4) level categories stratified by potential modifiers. **(A)** Model adjusted for fetal sex, prepregnant BMI, parity, education levels, TPO-Ab status and insurance; **(B)** Model adjusted for maternal age, fetal sex, parity, education levels, TPO-Ab status and insurance; **(C)** Model adjusted for maternal age, fetal sex, prepregnant BMI, parity, education levels, and insurance; **(D)** Model adjusted for maternal age, fetal sex, prepregnant BMI, education levels, TPO-Ab status and insurance. Maternal FT4 categories: Normal FT4 (11.7–19.7 pmol/L); Low FT4 (<11.7 pmol/L); and High FT4 (>19.7 pmol/L). FT4, free thyroxine; TPO-Ab, thyroid peroxidase antibody; BMI, body mass index (calculated as weight in kilograms divided by height in meters squared); PTD, preterm delivery.

We further conducted subgroup analysis stratified by maternal age, pre-pregnant BMI, TPO-Ab status, and parity (**Table 3** and **Figure 8**). We found stronger associations of low FT4 in early pregnancy with PTD risk among those who had a younger maternal age (<35 years old), higher maternal pre-pregnancy BMI (≥24 kg/m^2^), TPO-Ab negative and nulliparity (**Table 4**). We also identified a stronger association of high FT4 in early pregnancy with PTD risk among those who had a lower maternal pre-pregnancy BMI (<18.5 kg/m^2^) and the association did not differ in other subgroups (**Table 4**).

**Table 3:** Hazard ratios of preterm delivery with different FT4 categories in subgroup analysis.

Variables	FT4 categories	No. of Events/Total No. (%)	HR (95% CI)^a^	P-value
Maternal age, y				
<35	Normal	2,378/52,957 (4.49)	1 (reference)	
	Low	97/1,452 (6.68)	1.42 (1.15 to 1.74)	<0.001
	High	63/1,119 (5.63)	1.32 (1.03 to 1.69)	0.03
≥35	Normal	571/9,270 (6.16)	1 (reference)	
	Low	50/612 (8.17)	1.29 (0.96 to 1.73)	0.09
	High	17/155 (10.97)	1.86 (1.15 to 3.02)	0.01
Pre-pregnancy BMI				
<18.5	Normal	437/9,329 (4.68)	1 (reference)	
	Low	8/159 (5.03)	0.99 (0.49 to 2.01)	0.99
	High	24/306 (7.84)	1.75 (1.16 to 2.64)	0.008
18.523.9	Normal	2,072/45,551 (4.55)	1 (reference)	
	Low	91/1,371 (6.64)	1.33 (1.08 to 1.65)	0.008
	High	49/911 (5.38)	1.23 (0.93 to 1.64)	0.15
≥24	Normal	440/7,347 (5.99)	1 (reference)	
	Low	48/534 (8.99)	1.46 (1.08 to 1.98)	0.01
	High	7/57 (12.28)	2.02 (0.96 to 4.27)	0.06
TPO-Ab				
Negative	Normal	2,633/55,597 (4.74)	1 (reference)	
	Low	129/1,737 (7.43)	1.40 (1.17 to 1.67)	<0.001
	High	66/1,116 (5.91)	1.32 (1.03 to 1.69)	0.03
Positive	Normal	316/6,613 (4.78)	1 (reference)	
	Low	18/327 (5.5)	1.07 (0.66 to 1.73)	0.79
	High	14/157 (8.92)	1.97 (1.15 to 3.38)	0.01
Parity				
nulliparity	Normal	2,088/46,170 (4.52)	1 (reference)	
	Low	84/1,214 (6.92)	1.39 (1.11 to 1.74)	0.003
	High	57/999 (5.71)	1.33 (1.02 to 1.73)	0.04
multiparity	Normal	861/16,057 (5.36)	1 (reference)	
	Low	63/850 (7.41)	1.28 (0.99 to 1.66)	0.06
	High	23/275 (8.36)	1.65 (1.09 to 2.51)	0.02

Gestational weeks for thyroid function screening were 9–13 weeks in early pregnancy. Low maternal FT4 defined as FT4 <11.7 pmol/L, normal maternal FT4 as FT4 between 11.7–19.7 pmol/L and high maternal FT4 as FT4 >19.7 pmol/L. Model adjusted for age, fetal sex, pre-pregnancy BMI, parity, education status, TPO-Ab status and insurance.

FT4, free thyroxine; TPO-Ab, thyroid peroxidase antibody; HR, hazard ratio, CI, confidence interval; BMI, body mass index (calculated as weight in kilograms divided by height in meters squared).

**Table 4:** Sensitivity analysis for association of FT4 with PTD.

FT4			No. of events/Total No. (%)	HR 95% CI	P-Value	
Exclude history of preterm delivery						
	Normal		2,746/61,516 (4.46)	1 [Reference]		
	Low		135/2,019 (6.69)	1.35 (1.13 to 1.60)		<0.001
	High		73/1,259 (5.8)	1.37 (1.09 to 1.73)		0.008
Exclude thyroid medication during pregnancy						
	Normal		2,893/61,201 (4.73)	1 [Reference]		
	Low		138/1,975 (6.99)	1.32 (1.11 to 1.57)		0.002
	High		72/1,224 (5.88)	1.32 (1.04 to 1.67)		0.02
Exclude HDP and GDM						
	Normal		2,227/52,534 (4.24)	1 [Reference]		
	Low		93/1,525 (6.1)	1.34 (1.09 to 1.66)		0.006
	High		62/1,084 (5.72)	1.41 (1.10 to 1.82)		0.007

Models were adjusted for age, fetal sex, pre-pregnancy BMI, parity, education levels, TPO-Ab status and insurance.

FT4, free thyroxine; HR, hazard ratio; CI, confidence intervals; PTD, preterm delivery; HDP, hypertensive disorders during pregnancy; GDM, gestational diabetes mellitus.

For sensitivity analysis, the associations between low/high FT4 and risk of PTD were still robust after exclusion of women with PTD history, taking thyroid medication during pregnancy, GDM, and HDP (**Table 4**).

## Discussion

To the best of our knowledge, our findings revealed a U-shaped association between continuous spectrum maternal FT4 and PTD for the first time, providing a better fit for real-world clinical data. Our study further indicated both low and high maternal FT4 concentrations as risk factors for PTD. Moreover, the positive association of isolated hypothyroxinemia with PTD was mainly driven by spontaneous PTD while women with overt hyperthyroidism were more susceptive to iatrogenic PTD. We also illustrated that the association of high FT4 or overt hyperthyroidism with iatrogenic PTD was partially mediated through the development of HDP.

Our results demonstrated that abnormal maternal FT4 concentrations might attribute to a higher risk of PTD. Maternal thyroid hormone concentrations in the first trimester are pivotal for fetal growth (24, 27) and neurodevelopment (5, 7, 28) when the fetus depends solely on maternal thyroid hormones *via* transplacental transition (29). However, epidemiological research has shown inconsistent evidence concerning mild alterations of maternal thyroid function during pregnancy and PTD (15–17). Furthermore, limited research has looked into the association of thyroid dysfunction with subtypes of PTD (16, 30, 31). Most observational studies studied maternal thyroid test abnormalities with widely different definitions. This type of categorization of continuous variables might obscure important information, and few studies have evaluated the risk of PTD across the full spectrum of maternal FT4 concentrations. Therefore, taking advantage of an intrinsically interpretable machine learning model, our study is the first to analyze the non-linear dose-dependent relationship between maternal FT4 and PTD. Our results demonstrated that both low and high maternal FT4 is associated with a higher risk for PTD—indicating a beneficial role of early maternal thyroid function screening to identify high-risk PTD in pregnant women. In addition, isolated hypothyroxinemia and overt hyperthyroidism were identified as risk factors for spontaneous and iatrogenic PTD, respectively. Therefore, these results might provide new evidence towards the significance of timely clinical management concerning women with isolated hypothyroxinemia and overt hyperthyroidism identified in the first trimester. Furthermore, these results also encourage future research toward early management to maintain maternal FT4 in an optimal range for the prevention of adverse pregnancy outcomes.

The mechanisms behind the U-shaped relationship between maternal FT4 and PTD might be explained *via* several potential pathways and our mediation analyses underlined a differentiated PTD pathogenesis mechanism of high and low maternal FT4. First, the full compensatory mechanisms needed to improve the maternal–fetal transfer of thyroid hormones might be absent in the placenta of patients with pathological maternal thyroid hormone deficiency during gestation (32). In addition, disrupted endocrine factors, namely, vasopressin, under maternal thyroid hypofunction (33), and the inflammation process at the maternal–fetal interface triggered by oxidative stress (34–36), might be both related to the early onset of spontaneous PTD. Moreover, maternal thyroid hormone deficiency could also lead to insufficient trophoblast cell invasion, which might further lead to abnormal placentation and PTD (12, 37, 38). Importantly, maternal hyperthyroidism may accelerate the degradation of proteins and lipids which results in chronic maternal caloric deficiency and further adversely affects fetal growth (39). Overt hyperthyroidism is a well-acknowledged risk factor for HDP (40–44) and current clinical management of HDP includes timely termination of pregnancy under the more severe form of the disease in need of iatrogenic PTD. Our data indicated that the subsequent development of HDP might act as a bridge between high FT4 or overt hyperthyroidism and iatrogenic PTD, while no mediation effect of HDP was identified in the association between low FT4 or isolated hypothyroxinemia and spontaneous PTD.

GAM is an intrinsically interpretable machine learning model to reduce the mean squared error for the exposure effect after adjustment, and circumvent the increase of the type I error for testing the exposure effect (20). With no assumption of a specific priori functional association (e.g., linearity) between maternal FT4 and PTD, GAM enables the exploration of a smooth, possibly non-linear association that is determined by the data rather than the modeler. Moreover, the time-to-event (for PTD) method and multivariable Cox proportional hazard models were further applied to analyze the association of abnormally high and low maternal FT4 concentrations with the timing of PTD. We also differentiated the subtypes of PTD (including spontaneous and iatrogenic PTD) and explored which subtype primarily attributed to the association between different maternal thyroid dysfunctions and PTD. Taking advantage of this method, we further proved that isolated hypothyroxinemia pregnant women might have an incremental risk of spontaneous PTD while women with hyperthyroidism were more susceptive to iatrogenic PTD. Lastly, we also identified a potential mediating effect of HDP on the association between high FT4 concentration and PTD—the mechanism of which should be further studied in future.

Regardless, this study also has several limitations. First, selection bias might be present because it was a cohort study based on a single center; hence, multiple obstetric centers should be considered in future. Second, because maternal thyrotropin receptor antibodies (TRAb) were not available in the current study, we could not fully exclude the impact of a small number of undiagnosed Grave’s hyperthyroidism (TRAb-positive) even though we excluded women with pre-existing thyroid disease and thyroid medication. Third, although a series of potential confounders were adjusted, the possibility of residual confounding could not be completely ruled out. For example, the blood and urine iodine were not assessed during the routine pregnancy examinations. Although the city of Shanghai is not an iodine-deficient area (45), we could not fully eliminate the influence of the iodine status. Finally, this is an observational study, and our findings are warranted to be validated by future large, multi-center randomized control trials.

In conclusion, there is a U-shaped dose-dependent relationship between maternal FT4 in the first trimester and PTD. The association between isolated hypothyroxinemia and PTD was mainly associated with spontaneous PTD while overt hyperthyroidism may be attributable to iatrogenic PTD. Therefore, the early monitoring of maternal thyroid function during pregnancy could be important to identify high-risk PTDs. Future research into early management to maintain maternal FT4 in an optimal range are warranted for the prevention of adverse pregnancy outcomes.

## Data Availability Statement

Data available on reasonable request. Requests to access the datasets should be directed to fanjianxia122@126.com.

## Author Contributions

YulZ: Conceptualization, Methodology, Investigation, Form analysis, Writing—Original draft preparation. YL: Methodology, Writing—Original draft preparation & Review & Editing. YuaZ: Writing—Original draft preparation & Review & Editing. YoZ: Project Administration. JF: Conceptualization, Resources, Writing—Reviewing and Editing, Supervision, Funding acquisition, data interpretation and revision. WW: Conceptualization, Methodology, Software, Data curation, Writing—Review & Editing, Supervision, Funding acquisition. All authors listed have made a substantial, direct, and intellectual contribution to the work and approved it for publication.

## Funding

This work was supported by the National Key R&D Program of China [grant number 2018YFC1004602], the National Natural Science Foundation of China [grant numbers 81974235], awarded to JF and the National Natural Science Foundation of China [grant numbers 81971392], the Shanghai Municipal Committee of Science and Technology [grant number 19ZR1462200] awarded to WW. These funding organizations had no involvement in the study design; in the collection, analysis and interpretation of data; in the writing of the report; and in the decision to submit the article for publication.

## Conflict of Interest

The authors declare that the research was conducted in the absence of any commercial or financial relationships that could be construed as a potential conflict of interest.

## Publisher’s Note

All claims expressed in this article are solely those of the authors and do not necessarily represent those of their affiliated organizations, or those of the publisher, the editors and the reviewers. Any product that may be evaluated in this article, or claim that may be made by its manufacturer, is not guaranteed or endorsed by the publisher.
